# Assessment and management of dry eye disease in the UK: standardising reality-based best practice

**DOI:** 10.1038/s41433-026-04375-7

**Published:** 2026-03-14

**Authors:** Bita Manzouri, Sajjad Ahmad, Sophie Harper, Sai Kolli, David Lockington, Michael O’Gallagher, Harminder Dua

**Affiliations:** 1 Queen’s Hospital, BHR University Hospitals NHS Trust, Romford, UK; 2 Moorfields Eye Hospital NHS Foundation Trust, London, UK; 3 UCL Institute of Ophthalmology, London, UK; 4 Moorfields/UCL NIHR BRC, London, UK; 5 Manchester Royal Eye Hospital, Manchester University NHS Foundation Trust, Manchester, UK; 6 Queen Elizabeth Hospital, University Hospitals Birmingham NHS Trust & Aston University, Birmingham, UK; 7 Tennent Institute of Ophthalmology, Glasgow, UK; 8 Royal Victoria Hospital, Belfast, UK; 9 Academic Ophthalmology, Mental Health and Clinical Neurosciences, School of Medicine, University of Nottingham, Nottingham, UK

**Keywords:** Corneal diseases, Diagnosis

## Abstract

**Background:**

Assessment and management of dry eye disease (DED) in the UK is increasingly taking place outside of specialist ophthalmology settings. While comprehensive, evidence-based international guidance exists, much of it does not reflect the realities of practice in the UK. A panel of experts was brought together to identify areas of consensus on assessment, management, and appropriate referral of DED in the UK National Health Service (NHS).

**Methods:**

A questionnaire was circulated to a panel consisting of 15 optometrists, ophthalmologists, and corneal specialists with experience and expertise in DED. Based on their responses, consensus statements were developed and underwent two rounds of voting, in which respondents indicated to what extent they agreed with each statement. A core steering panel of seven experts discussed the results and provided further context for the statements.

**Results:**

Strong or very strong consensus was reached for 57/62 statements. Statements with very strong consensus included guidance on the minimum symptoms and signs to be assessed on initial presentation and simple guidance for grading the severity of the disease. Statements regarding initial treatment were divided by setting (primary and secondary care), and a strong or very strong consensus was reached on 17/20 statements relating to treatment options in these settings. Statements specific to referral included approximate target timelines, where possible, as well as guidance on key supporting information to help improve the efficiency of patient care.

**Conclusions:**

This consensus provides a UK-focused resource to support consistent and effective care for patients with DED within the NHS.

## Introduction

Dry eye disease (DED), a multifactorial disease of the ocular surface involving loss of tear film homoeostasis and ocular surface inflammation [1, 2], can result in symptoms including discomfort and changes to vision [1, 3, 4]. DED affects around one third of the UK population [5]. The impact of DED on patients’ quality of life (QoL), both mental and physical, can be substantial [3, 4, 6–8], and the experience of moderate or severe disease has previously been compared to that of angina [9]. Furthermore, DED is associated with a substantial impact on productivity and work life, even for mild or moderate disease [8], with an increased risk of unemployment and a rate of absenteeism above the average [10]. Assessment of DED is complicated by the fact that signs and symptoms do not always correlate; notably, patients can present with debilitating pain associated with few clinical signs (termed neuropathic pain) [2, 4, 11].

Global guidance on the assessment and management of DED was developed by the Tear Film and Ocular Surface Dry Eye Workshop II (TFOS DEWS II) [1, 12, 13] and updated recently as DEWS III [2, 8, 14]; but international guidelines, however comprehensive, cannot fully reflect the realities of working within the UK National Health Service (NHS). DED in the UK is increasingly being managed outside of specialist corneal services, often in community optometry, sometimes by independent prescribing (IP) optometrists [15], or in hospital general ophthalmology clinics. Clinicians have variable degrees of experience in the work-up and treatment of patients with DED and the availability of equipment and ocular medications is inconsistent across the UK. While NHS guidance is available [16, 17], there is a lack of granular detail, particularly with respect to the information that should be captured and communicated along the patient pathway.

With a scarcity of detailed, localised guidance, there is broad variation in the assessment and management of DED [15] by both geographical location and clinical setting (primary versus secondary or tertiary care). This variation can lead to incomplete testing or tests being repeated unnecessarily and, at times, inadequate or delayed treatment.

Ophthalmology is currently the busiest outpatient speciality in the country, with over 588,000 patients waiting for consultant-led ophthalmology treatment at the end of April 2025: this constitutes 8% of the entire NHS elective backlog [18]. These numbers present the prospect of long waits for referrals to secondary or tertiary care while patients struggle with sometimes-debilitating symptoms. Further, patients frequently present at acute ophthalmic services in secondary care with non-urgent issues that should be manageable in the community [19]. UK-specific guidance, identifying ways to streamline patient pathways, maximise community treatment where possible, and to optimise the process of referral where necessary, may help to reduce the strain on Hospital Eye Services (HES) capacity and ensure patients receive optimal care.

A panel was convened to critically consider assessment, treatment and referral of DED in the UK. The panel, comprising a steering committee of leading experts and a wider group of ophthalmologists representing various geographical areas across the country, followed a Delphi-adjacent process to formulate key recommendations for best-practice management of DED in the UK. These recommendations are intended to support community and hospital-based optometrists and ophthalmologists to diagnose, manage and refer adult patients with DED in a consistent and thorough manner.

## Materials and methods

### Expert panel

A steering committee (SC) of seven UK experts in DED led the consensus process, while a further nine ophthalmology consultants, fellows and senior trainees in the field from across the country were recruited to support the process as part of the expert panel (see Supplementary Table 1). The steering group members were recruited on the basis of their extensive experience managing DED in NHS secondary or tertiary centres in a mix of settings (large teaching hospitals and district hospitals). The broader panel members were recruited from a wider range of experience levels to capture day-to-day management in a range of settings. Overall, the panel members have an average (mean) of 13 years of experience in the field, and each see between 200 and 1500 patients with DED each year.

### Process

As a first step, a questionnaire of 66 questions with largely free-text responses was circulated to collect information on the current clinical practices and experience of all the panel members, including assessment, diagnosis, grading, treatment, referral, follow-up and discharge. Based on the free-text responses to these questions, a second questionnaire was developed containing 117 draft statements and 20 ranking and prioritisation exercises across these categories. A patient pathway was also developed based on the panel’s responses to reflect the patient experience with DED in the UK (Supplementary Fig. 1). The panel members rated their level of agreement or disagreement with each statement; responses were collated and a consensus score calculated, as below. Consensus was achieved on some statements at the first round of voting; those with differing opinions were discussed on a follow-up call and subsequently re-phrased or clarified. A final list of 64 refined statements was circulated to the full panel, with voting options for scoring as below. Two statements were subsequently removed from the list as their phrasing was considered too ambiguous. The process broadly followed a Delphi-adjacent methodology, but with additional discussion in order to help find agreement on areas with weaker consensus.

### Definition of consensus

Each voting option was assigned a score: ‘strongly agree’ scored 4 points, ‘slightly agree’ 2 points, ‘don’t know/not applicable’ 0 points, ‘slightly disagree’ –2 points and ‘strongly disagree’ –4 points. Based on the votes received and their respective scores, a weighted average score was calculated (with a maximum possible score of 4) and this and the percentage of respondents choosing ‘strongly agree’ factored into the calculated strength of consensus:

- Very strong consensus: score ≥3, ‘strongly agree’ picked by >70%
- Strong consensus: score >2, >50% strongly agree and/or >80% agree to some extent
- Moderate consensus: score ≥2, >70% agree to some extent
- Weak consensus: score <2 but positive, ≥60% agree to some extent
- No consensus: score <2, <60% agree to some extent

## Results

In total, 15 votes were received on the final set of consensus statements. A moderate-to-strong consensus was reached in the majority of cases (see Table 1 and Supplementary Table 2), which fell into eight categories: initial assessment, DED subtypes, grading severity, initial treatment in the community, initial treatment in secondary care, referral, ongoing management and follow-up, and discharge. Recommendations are per patient unless otherwise indicated.

**Table 1:** Key consensus statements and scores.

Statement		Consensus score	Percentage who *strongly agree*	Percentage who *strongly agree* or *slightly agree*	Strength of agreement
***Initial assessment***					
1	In order to diagnose DED, it is essential to capture information on both symptoms and signs*	**3.87**	**93.3**	**100.0**	**Very strong**
2	It is important to ask an open question about the patient’s symptoms, but to follow up on, as a minimum, **pain,** **grittiness** (or foreign body sensation), **blurred vision,** **photophobia** and **redness**	**3.73**	**86.7**	**100.0**	**Very strong**
3	Symptomatic assessment, in every setting, should include timing and triggers of symptoms as well as their duration, nature, and impact on quality of life	**3.73**	**86.7**	**100.0**	**Very strong**
4	In an ideal world, symptom questionnaires can offer an objective baseline for ongoing management, but barriers in terms of both time and practical considerations limit the feasibility of their use	**2.93**	**60.0**	**93.3**	**Strong**
5	The presence of **dry mouth** (and/or dry **nostrils**) may be suggestive of Sjogren’s Syndrome and should be captured if present	**3.73**	**86.7**	**100.0**	**Very strong**
6	As a minimum, the following signs should be assessed* on initial patient presentation: **tear break-up time, fluorescein staining** (including for PEEs), **conjunctival injection/hyperaemia** and **eyelid assessment** (including MGD)	**4.00**	**100.0**	**100.0**	**Very strong**
8	Corneal sensation testing should be performed as standard* if a patient reaches secondary care in order to rule out corneal neuropathy	**3.47**	**73.3**	**100.0**	**Very strong**
9	Where a Schirmer’s test (unanaesthetised) can be competently performed, this can provide useful additional information but is not required for a basic assessment	**2.67**	**46.7**	**93.3**	**Moderate**
10	When assessing a patient with suspected DED, it is essential to ask about ocular co-pathology and medications, along with any past ocular procedures and prior or current measures taken to address their DED symptoms	**3.87**	**93.3**	**100.0**	**Very strong**
***Grading the severity of DED***					
16	The presence of DED can be inferred from a combination of fluorescein staining and short ( ≤ 10 s) tear break-up time (TBUT)	**3.20**	**60.0**	**100.0**	**Very strong**
17	**Severe** DED can be indicated (in either eye) by an Oxford staining score of 4 or greater, along with a TBUT of 3 or fewer seconds and a substantial negative impact on the patient’s quality of life; filamentary changes can also be indicative of severe disease	**4.00**	**100.0**	**100.0**	**Very strong**
18	**Moderate** DED can be indicated (in either eye) by an Oxford staining score of 2–3, along with TBUT of 4–6 s and a moderate impact on the patient’s quality of life	**3.73**	**86.7**	**100.0**	**Very strong**
19	**Mild** DED can be indicated (in either eye) by an Oxford staining score of 0–1, along with a TBUT of 7–10 s and a mild impact on the patient’s quality of life	**3.60**	**80.0**	**100.0**	**Very strong**
20	If a patient lacks staining but is experiencing pain (neuropathic pain) in either eye^†^, they should be referred to an appropriate specialist	**3.33**	**80.0**	**93.3**	**Very strong**
***Initial treatment of DED in the community***					
21	Initial treatment in the community setting should comprise lid hygiene and lifestyle measures, along with unpreserved lubricants*	**3.87**	**93.3**	**100.0**	**Very strong**
22	Patients with mild DED and no other concerns should be managed by a community optometrist	**3.71**	**85.7**	**100.0**	**Very strong**
25	A range of lubricants are available for treatment of DED, but any lubricant used must be preservative-free	**2.40**	**53.3**	**86.7**	**Strong**
26	A once-daily administration of lubricants is unlikely to be sufficient: patients should be advised to apply them at least 3–4 times daily*	**3.33**	**66.7**	**100.0**	**Very strong**
28	If symptoms are not controlled despite frequent use of lubricant drops, escalation of treatment should be considered	**3.60**	**80.0**	**100.0**	**Very strong**
***Initial treatment of DED in secondary care (general ophthalmology)***					
32	As a minimum, assessments on presentation in secondary care* should include fluorescein staining, TBUT, vision, lid function and blink reflex, tear meniscus height and meibomian gland assessment	**3.47**	**86.7**	**93.3**	**Very strong**
33	A simple assessment of corneal sensation (presence or absence)* should also be performed in secondary care	**3.73**	**86.7**	**100.0**	**Very strong**
34	The tests outlined above (minimum assessments on presentation in secondary care) should be repeated even if results from prior testing are available, in case of changes	**3.87**	**93.3**	**100.0**	**Very strong**
35	The following treatments can be considered for prescription by a general ophthalmologist: short-course topical corticosteroids, ciclosporin A, oral doxycycline, acetylcysteine 5%	**3.87**	**93.3**	**100.0**	**Very strong**
36	The following treatments should be prescribed only by a corneal specialist: scleral lenses, insulin, secretagogues, autologous serum eyedrops, oral pilocarpine, off-licence agents	**3.47**	**86.7**	**93.3**	**Very strong**
38	If a patient responds positively to steroids, indicating that inflammation is present, treatment with ciclosporin A should be initiated to minimise steroid-related side-effects	**3.47**	**73.3**	**100.0**	**Very strong**
40	Punctal plugs can be considered once inflammation is controlled but should not be initiated beforehand	**3.60**	**80.0**	**100.0**	**Very strong**
***Referral***					
41	Severe corneal damage (corneal melting, thinning or perforation or confluent SPK), a corneal ulcer or (super)infection should trigger an **urgent** referral to eye casualty, or a corneal specialist dependent on local pathways	**3.87**	**93.3**	**100.0**	**Very strong**
42	Patients with atypical or neurotrophic signs (such as a persistent defect of the cornea or other features that would heighten anxiety about a patient) should be referred **soon** to a corneal specialist	**4.00**	**100.0**	**100.0**	**Very strong**
44	Patients with an underlying systemic or autoimmune disease, unexplainable changes in vision, or recent-onset redness that cannot be resolved with over-the-counter medicines or antibiotics, should be referred to a general ophthalmologist or corneal specialist in a **routine** timeframe	**2.27**	**53.3**	**80.0**	**Strong**
45	Patients with severe DED that is refractory to treatment should be referred to a corneal specialist in a **routine** timeframe	**2.93**	**73.3**	**86.7**	**Strong**
46	Timeframes for referrals may vary according to local resources, but **urgent** referrals should ideally be within 24–48 h, **soon** referrals within one week to one month and **routine** referrals within several months	**3.73**	**86.7**	**100.0**	**Very strong**
47	Referral letters from general ophthalmology to cornea should always include, as a minimum, information about: vision, current medications, prior surgeries, prior DED treatments, advice and outcomes, corneal fluorescein staining score, red-flag signs, any systemic diseases, tear meniscus height, meibomian gland function assessment (blepharitis, anterior, posterior), lid function, and corneal sensation	**2.53**	**60.0**	**86.7**	**Strong**
48	If a patient who has previously been successfully treated with short-course, mild steroids experiences a flare-up, it is advisable for a community prescriber to initiate a new short course while waiting for a referral to avoid deterioration	**2.27**	**46.7**	**86.7**	**Moderate**
***Ongoing management and follow-up***					
49	Frequency of follow-up is dependent on the level of disease control, as defined by the tolerability of the patient’s symptoms and degree to which signs are resolved	**3.87**	**93.3**	**100.0**	**Very strong**
50	DED is considered to be under control when symptoms are reduced by treatment to a level the patient considers tolerable and signs have improved from baseline, with no clinical evidence of inflammation	**3.73**	**86.7**	**100.0**	**Very strong**
55	Treatment for DED is generally of a long-term nature, requiring repeat prescriptions: it is important that GPs are aware of this need	**3.87**	**93.3**	**100.0**	**Very strong**
***Discharge***					
56	A patient with DED can be discharged when symptoms are stable and well-controlled with no need for specialised treatments, and when they are both happy and able to self-manage	**3.87**	**93.3**	**100.0**	**Very strong**
59	The patient’s GP and/or community optometrist should be copied into a letter explaining previous and ongoing management of the disease	**3.87**	**93.3**	**100.0**	**Very strong**
60	Patient-initiated follow-up (PIFU) could be offered for a period of time from 12–36 months depending on the initial grade of severity of DED and on local capacity	**3.6**	**80.0**	**100.0**	**Very strong**

Additional consensus statements are included in Supplementary Table 2.

*Statement applies to both eyes. ^†^In the panel’s clinical experience, neuropathic pain is generally found in both eyes.

*CsA* ciclosporin A, *MGD* meibomian gland dysfunction, *PPE* punctate epithelial erosions, *PIFU* patient-initiated follow-up, *SPK* superficial punctate keratitis.

### Initial assessment

While many patients with DED may attempt to manage their condition alone, with or without input from a GP or pharmacist [20], the scope of this consensus relates to their contact with eyecare professionals, either in the community or the hospital setting.

**Fig. 1:**
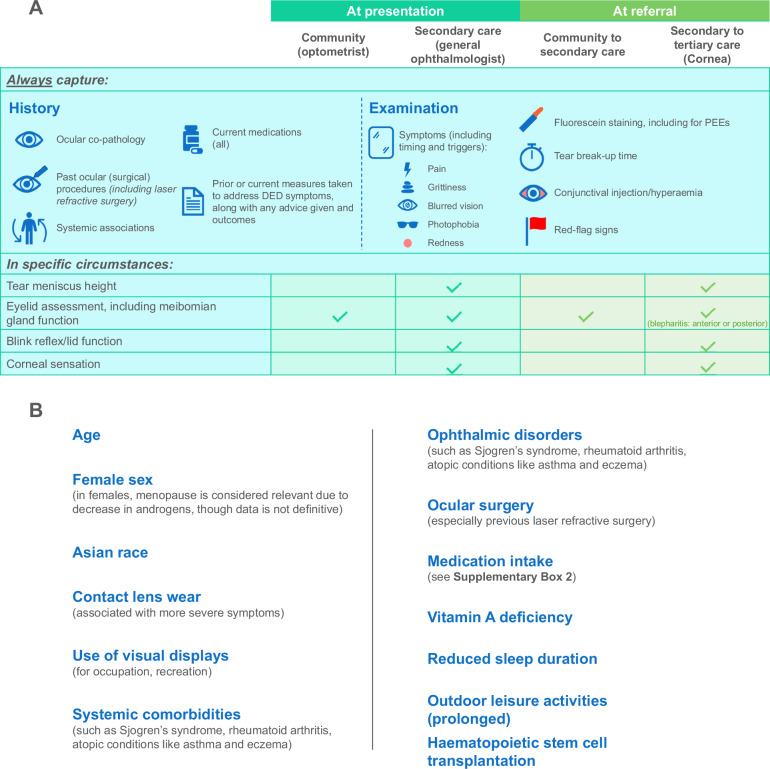
Minimum information to be captured and risk factors to consider when assessing DED. **A** Minimum information to capture in each setting (assessment and referral). Information that should always be captured, regardless of setting, is divided into history taking and examination. Below, information that is considered essential in some settings is listed with the relevant clinical settings indicated. **B** Risk factors for DED. Sources: Stapleton F, *et al. Ocul Surf* 2017;15:334–365; Stapleton F, *et al. Am J Ophthalmol* 2025; 279:451–553; Sullivan DA, *et al. Ocul Surf* 2017;15:284–333; Vidal-Rohr M, *et al. Cont Lens Anterior Eye* 2023;46 (3):101837.

A strong or very strong consensus was reached on most aspects of initial assessment (Table 1 and Supplementary Table 2, statements 1–12). Because assessment of both symptoms and signs is necessary for a diagnosis of DED [2, 15, 16] (*very strong consensus*), a strong consensus was reached that DED cannot be diagnosed without the use of a slit lamp. There was a very strong consensus on the minimum specific symptoms and signs that should be documented as part of the initial assessment (see Fig. 1A), as well as the importance of capturing the timing, triggers, duration and impact of the patient’s symptoms, and questions around ocular co-pathology, medications and prior surgical procedures. In addition, a very strong consensus was reached on the need to capture symptoms such as dry mouth or dry nostrils, as these may be suggestive of Sjögren’s syndrome (statement 5). A strong consensus was reached that symptom questionnaires such as the OSDI [2, 21, 22] are potentially useful to provide an objective baseline of the patient’s disease, but there can be practical barriers to their use.

Notably, only a moderate consensus was reached on use of the Schirmer’s test (unanaesthetised eye, Schirmer-1 test) as part of an initial assessment. The vast majority (93.3%) of the panel agreed with this statement to some extent, but a paucity of high-level evidence on its reliability, sensitivity and specificity [12], as well as the time required and variability of experience and skill levels of those conducting the test, may contribute to the difficulty in finding very strong consensus on this point.

The importance of asking female patients about hormonal medications or supplementation, due to the known link between sex hormones and DED [8, 23, 24], reached a moderate consensus, as did the consideration of referral to secondary care for patients with connective tissue disorders (see Supplementary Table 2). Risk factors for DED, and systemic medications known to be linked to the disease, are listed in Fig. 1B and Supplementary Box 1.

Red-flag signs that reached consensus from the panel include severe corneal damage (such as corneal melting, thinning or perforation or confluent superficial punctate keratitis; illustrative images of the latter are included for reference in Supplementary Figure 2), corneal ulcer, infection or superinfection, atypical or neurotrophic signs; these should be triggers for urgent referral (*very strong consensus*) (see *Referral* section).

### DED subtypes

There was a very strong consensus on all three statements (Supplementary Table 2, statements 13–15) surrounding the DED subtypes: evaporative DED (EDED), aqueous-deficient DED (ADDED) and mixed-type DED. Although treatment of each subtype can be slightly different, owing to their different aetiology, in general most cases include elements of both; an evaporative component is considered more common than an aqueous component [2, 25].

### Grading severity

**Fig. 2:**
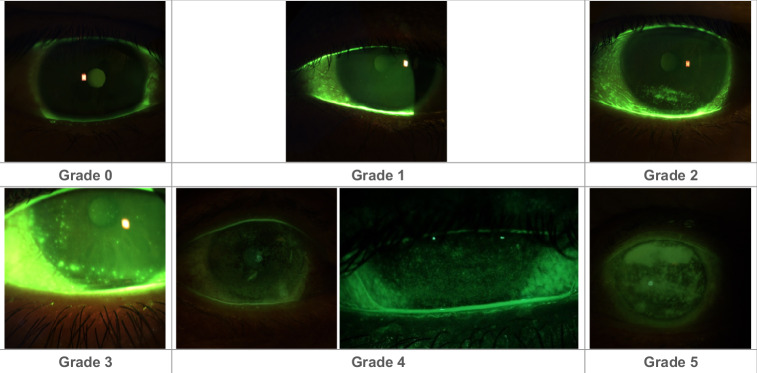
The Oxford grading scale. Illustrative images of eyes scoring 0 to 5 on the Oxford grading scale are shown to aid with grading. Images were provided by Professor Sai Kolli.

All statements regarding grading the severity of DED (Table 1, statements 16–20) reached very strong consensus. Guidance on applying the Oxford grading scale [26] is provided in Fig. 2. In all cases, the severity of the impact on the patient’s quality of life should be taken into account when grading the disease. Patients experiencing ‘pain without stain’ (neuropathic pain [11]) should be managed in collaboration with an appropriate non-ophthalmic specialist (*very strong consensus*), for example a pain specialist.

### Initial treatment in the community

**Fig. 3:**
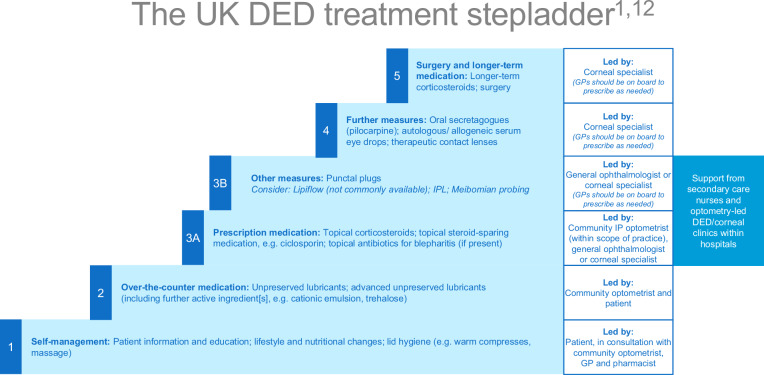
The UK DED treatment stepladder. Guidance on who should be responsible for leading care is included in boxes above each step. *IPL* intense pulsed light. Sources: Craig JP, *et al. Ocul Surf* 2017;15(4):802–812; Wolfssohn JS, *et al. Ocul Surf* 2017;15(4):539–574.

The panel reached a very strong consensus on most statements on initial treatment in the community (Table 1 and Supplementary Table 2, statements 21–31), including first-step treatments, self-management and the importance of education and management, where possible, by a community optometrist. A UK-centric DED treatment stepladder was developed, based on the DEWS II stepladder [13] and adjusted in response to feedback received (Fig. 3). The panel reached a strong consensus that any lubricant used must be preservative-free, the level of consensus perhaps limited by conflicting evidence in the literature [27–30], but very strongly agreed that they should be applied 3–4 times daily, since a once- or twice-daily administration is unlikely to be sufficient [30]. A very strong consensus was reached on the treatment of patients with EDED, who may require a greater focus on lid hygiene and lipid-containing artificial tears. Notably, the panel was unable to reach a strong consensus on the initiation of topical corticosteroids and ciclosporin A (CsA) in the community setting by an IP optometrist. This may be because of the current limited formulary for IP optometrists.

### Initial treatment in secondary care

This section covers both initial assessment and treatment on presentation to secondary care (Table 1 and Supplementary Table 2, statements 32–40). There was a very strong consensus that on presentation to secondary care, initial assessments should comprise assessment of lid function and blink reflex [31] as well as the tear break-up time (TBUT), fluorescein staining, vision, tear meniscus height and meibomian gland assessment—even if results are available from prior testing (*very strong consensus*). A simple assessment of corneal sensation is also advised (*very strong consensus*). The minimum recommended information to capture on presentation to secondary care is summarised in Fig. 1A.

A very strong consensus was reached on most forms of treatment in secondary care, including specific treatments for subtypes of DED and whether selected therapies can be prescribed by a general ophthalmologist or should be left to the discretion of a corneal specialist. However, there was weak consensus on who should prescribe topical azithromycin, with only 60% agreeing strongly or slightly that this should be the corneal specialist. Consensus on the steroid-sparing use of CsA in patients who respond positively to steroids was very strong.

### Referral

This section included both timelines for referral and processes (Table 1 and Supplementary Table 2, statements 41–48). There was very strong consensus on most signs that should trigger ‘urgent’, ‘soon’ or ‘routine’ referral to eye casualty, a corneal specialist or a general ophthalmologist (see Table 2). Urgent (known in some settings as ‘emergency’) referrals were specified as being within 24–48 h, ‘soon’ referrals within one week to one month and routine referrals within several months (*very strong consensus*). However, only weak consensus was reached on the referral of patients with filamentary keratitis, with some respondents preferring direct referral to a corneal specialist. A moderate consensus was reached regarding the community initiation of a short course of corticosteroids for patients who have previously been successfully treated and have experienced a flare-up.

**Table 2:** Ideal referral timelines and recommended referral pathway.

Timeline	Issue	Referral to:
**Urgent**	Corneal melting	Eye casualty/corneal specialist
**Urgent**	Signs of infection or ulceration	Eye casualty/corneal specialist
**Urgent**	Corneal thinning with impending perforation	Eye casualty/corneal specialist
**Soon**	Confluent SPK	Eye casualty/corneal specialist
**Soon**	Filamentary keratitis	General ophthalmology
**Routine**	DED refractory to treatment	Corneal specialist
**Routine**	Systemic associations	General ophthalmologist (or corneal specialist if additional red flags are present)

Note: Timeframes for referrals may vary according to local resources. Terminology may vary locally: for the purposes of this article, **urgent** referrals should ideally be within 24–48 h, **soon** referrals within one week to one month and **routine** referrals within several months.

### Ongoing management and follow-up

A very strong consensus was reached on general statements around frequency of follow-up, long-term management and the definition of disease control for DED (Table 1 and Supplementary Table 2, statements 49–55). Detailed recommendations on frequency of follow-up are covered in Supplementary Table 3. The statement that lubricants and lid hygiene should be continued for life but tailored to the patient’s needs reached moderate consensus, while the panel only weakly agreed that doxycycline should be held in reserve for rescue if symptoms flare up, with less than a third of respondents strongly agreeing.

### Discharge

A strong to very strong consensus was reached on all statements regarding discharge (Table 1 and Supplementary Table 2, statements 56–62). There was strong consensus that patients who had previously been treated for moderate DED should initially present to their community optometrist if they are not in a patient-initiated follow-up (PIFU) scheme (*strong consensus*).

## Discussion

This article provides a UK-focused consensus on the assessment, management and referral of DED, with the aim of providing comprehensive, aligned and localised guidance for UK community and hospital-based optometrists and ophthalmologists. The panel was able to reach a moderate-to-strong consensus on almost all statements, with those relating to IP optometrists forming a notable exception. IP optometrists are able to prescribe licensed therapies, including pharmacological management, for ocular conditions including DED [15, 32]. They should work within their area of expertise and with support and supervision as appropriate [33]; not all IP optometrists may be confident and competent to prescribe topical corticosteroids or steroid-sparing anti-inflammatory agents like CsA, but a UK study found that when appropriately trained, their diagnosis and management decision-making in an acute hospital setting is equivalent to that of consultant ophthalmologists [19]. Within HES across the UK, a scope-of-practice survey found that IP optometrists prescribe medications required by patients following an in-clinic optometry assessment more frequently than general practitioners (GPs) [34]. Optometrists are also playing an increasingly central role as the first point of contact for eye care provision across the UK [15], but there is variation between and within the nations (England, Northern Ireland, Scotland and Wales) [15, 35–37]. Previous studies have shown that, with IP optometrists integrated into primary care pathways, very few patients require hospital referral [37]. There is the potential for IP optometrists to play a more significant and consistent role in the treatment of moderate-to-severe DED across all nations of the UK.

Statements concerning initial assessment generally achieved strong consensus, but a few only reached moderate levels of agreement. Symptom questionnaires are widely used in clinical studies of DED [2, 22] and can provide an objective symptomatic baseline to assess the progress of the patient on treatment; indeed, the OSDI-6 is specifically recommended in the recently published DEWS III [2]. However, in the daily clinical setting in the NHS, time in particular is a significant barrier and these questionnaires are not widely used [15]. In discussions, questions were also raised about how symptom questionnaire results would be communicated further along the patient pathway, given the lack of a universally integrated approach to electronic patient records [38]. Until these issues can be addressed, it is unlikely that symptom questionnaires will become a standard part of clinical assessment for dry eye disease in the UK.

The panel could only reach moderate consensus on the role of the Schirmer’s test. While it can provide useful information—particularly where ADDED is suspected – its reliability as a measure of DED is debated, particularly for mild forms of the disease [39, 40]. Additionally, Schirmer’s strips are not universally available in community practice and the test takes a long time (5 min [39]), making it impractical to position as an essential test in the context of busy NHS eye clinics. There are numerous additional signs and assessments that are not covered in the statements within this article, including osmolality and meibography; some of these require specialist equipment and others are controversial. For reference, an overview of the strengths and weaknesses of these techniques is included in Supplementary Table 4. In general, the panel felt it was important to focus on what would form an acceptable minimum standard that could be achieved in all settings, which is reflected by the small number of recommended assessments. The recently published DEWS III guidance strongly recommends the use of conjunctival staining with lissamine green [2], a test that is not readily available in the UK—particularly in community settings. As this test is therefore not practical within NHS practice, it was not included as an essential assessment by the panel. Additionally, corneal sensation testing is an important assessment that should take place at some point to rule out serious neurological damage, but the panel recognises it is not possible to conduct objectively in most settings.

There was a lack of strong consensus that female patients should be asked about hormonal medications, such as birth control or HRT. This is surprising, given sex hormones and in particular HRT and anti-androgens are thought to be risk factors for DED [8, 23, 24], and female sex is in itself a risk factor [8, 24] (see Fig. 2 and Supplementary Box 1). A stronger consensus may have been reached if the statement referred specifically to female patients over the age of 40 or around the age of menopause.

When assessing patients who have connective tissue disorders, only 40% of respondents strongly agreed that they would consider such patients for hospital referral if they experienced symptoms of DED; this may have achieved stronger consensus if the focus of the statement had been on the systemic symptoms, rather than DED itself. The link between DED and connective tissue disorders such as rheumatoid arthritis is well established and has been suggested to result from immune cell infiltration and associated inflammation in ocular tissues [8, 23, 41], as well as potentially from medications for rheumatological disorders [23].

Patients undergoing treatment in the community whose DED cannot be controlled with multiple applications of artificial tears, or whose DED has a significant evaporative component, may be advised to try more advanced lubricants: those containing more than one active ingredient, such as lipids [42] or trehalose. These classes of artificial tears may help to stabilise the tear film, as well as lubricating the ocular surface [43]. The relatively low proportion of the panel (53%) who strongly agreed on the necessity of preservative-free artificial tears was surprising, given the general preference in the treating community for preservative-free options [8, 15, 42]. Some reviews have recently suggested there is a lack of strong evidence for this preference [27, 29], which may have influenced the outcome here, as well as the availability of new, less toxic preservatives [8].

It is expected that assessments in secondary care will be more comprehensive than those carried out in the community setting, both as a result of available equipment and because patients presenting to secondary care are more likely to have complex or severe issues. For dry eye disease, assessment of lid function should include ensuring the blink reflex is complete, as well as investigating for the presence of lagophthalmos, entropion and ectropion. Statements around treatment in secondary care generally reached strong or very strong consensus, with the exception of topical azithromycin, which only a little over half of the panel agreed should be prescribed by a corneal specialist. In discussions, prescribing of azithromycin varied in dosing and frequency even within the core steering group.

Secondary care treatments not discussed in detail by the panel included meibomian orifice probing, intense pulsed light (IPL) and Lipiflow: these are not commonly available on the NHS. However, a brief overview of these treatments is included in Supplementary Box 2, to support discussions with patients who enquire about them following their own research.

Red flags that should trigger urgent or emergency referral were identified by the panel as severe corneal damage (such as corneal melting, thinning or perforation or confluent superficial punctate keratitis), corneal ulcer, infection or superinfection, atypical or neurotrophic signs. These align with severe complications identified in the NICE Clinical Knowledge Summaries [16] as requiring urgent referral: punctate epithelial erosions of the conjunctiva and cornea; corneal scarring, thinning, ulceration, or neovascularisation; corneal infection; corneal perforation (rare) and severe visual loss (rare). The Clinical Knowledge Summaries also highlight additional red-flag symptoms (sudden-onset pain or visual loss, persistent or severe visual loss, diplopia, unilateral symptoms, or systemic symptoms such as weight loss or fever) necessitating a same-day assessment [16].

The panel reached weak consensus that filamentary keratitis, a potential complication of DED that can cause pain and discomfort [44], should be cause for referral to secondary care in a routine timeframe (within several months). Some respondents preferred referral to a corneal specialist and the timeframe in the statement may also have contributed to the poor consensus: a ‘soon’ timeframe would perhaps have received more support, and this is shown in Table 2.

A patient who previously underwent successful treatment with short-course, mild topical steroids may experience a flare-up later on. Owing to the time needed for referral, and the sometimes-debilitating nature of symptoms even when an urgent referral is not required, a moderate consensus was reached that where non-potent steroids have been successful before, a new course could be initiated by a community prescriber in the meantime, applying only to low-potency topical corticosteroids (commonly including fluorometholone, prednisolone phosphate and hydrocortisone sodium phosphate [45]); this is a pragmatic recommendation to avoid further deterioration of the patient’s condition.

While very strong consensus was reached on most statements around follow-up and ongoing management, there was weak consensus that doxycycline should be held in reserve for rescue if symptoms flare up. Doxycycline, a member of the tetracycline family of antibiotics, has anti-inflammatory properties [46] and is commonly part of treatment for MGD [14, 47]. However, evidence around its use in DED is mixed [14, 47] and the optimal dosing of doxycycline, along with other tetracyclines, has not been well established [14]. The panel’s opinions on its routine use may also be influenced by concerns around potential gastrointestinal side effects, photosensitivity, and significant risks to the child in pregnant and breastfeeding mothers [48].

Strong consensus was reached that the frequency of follow-up is dependent on the level of disease control. Supplementary Table 3 outlines the frequency of follow-up recommended dependent on the patient’s status, from weekly for those with ongoing red-flag or urgent issues, to six-monthly for patients whose DED is responding to treatment with no or manageable side-effects. These latter patients should be considered for discharge to their local optometric practitioner.

In general, strong or very strong consensus was reached on all statements relating to discharge. Important points include education for patients to support ongoing self-management, communication with community practitioners on discharge from secondary care, ensuring continuity of treatment even once the patient is no longer involved with the hospital eye service, and the role of PIFU, where possible.

This study, as a qualitative consensus exercise, does have limitations, including the process adopted for selection of representation on the panel. Furthermore, geographical distribution was limited, with no representation from specialists based in Wales, and only one member of the steering panel is an IP optometrist. The conclusions of the panel members may not therefore be equally applicable to all devolved nations of the UK, which have differing healthcare systems. The process deviated from a Delphi approach by incorporating multiple rounds of verbal discussion, in order to help refine statements further; these discussions were by definition not anonymous, although rounds of voting were recorded anonymously.

In summary, this article offers a consensus view on management of DED specific to the UK. The key consensus recommendations may help to support nationwide consistent and effective assessment, diagnosis, management and referral, irrespective of clinical setting. For future directions, there is a plethora of devices and equipment now available for diagnosing, objectively documenting, and treating DED. A consensus on what might be the most appropriate additional modalities, both effective and affordable within the NHS setting, would be valuable. This could then form the basis for recommendations to NHS management to support making this technology available across the DED patient pathway.

## Summary

### What was known before

- Global guidance on the assessment and management of DED is available, but international guidelines cannot fully reflect the realities of working within the UK National Health Service
- Assessment, management and referral of DED is variable across the UK owing to differences in the settings in which it is managed, as well as the experience levels and expertise of those managing it
- Comprehensive UK-specific guidance, aimed at non-specialists (optometrists and general ophthalmologists), was lacking prior to the development of this article

### What this study adds

- This article includes statements on topics ranging from assessment and referral to management and discharge of patients with DED, in both the primary and secondary care settings
- These statements, developed and voted on by a panel of experts in the field, may offer guidance that can help to reduce the strain on hospital eye service capacity and ensure patients receive consistent and optimal care

## Supplementary information

Supplementary Table 1

Supplementary Table 2

Supplementary Table 3

Supplementary Table 4

Supplementary Box 1

Supplementary Box 2

Supplementary Figure 1

Supplementary Figure 2

## Data Availability

Data sharing is not applicable to this article as no datasets were generated or analysed during the current study.
