# Supplementary material for: Assessment and management of dry eye disease in the UK: standardising reality-based best practice
Source: Eye (Lond). 2026 Mar 14;40(8):1185–95. doi: 10.1038/s41433-026-04375-7 (PMC13195173; doi:10.1038/s41433-026-04375-7)
Supplement: Supplementary file 1 — Supplementary Table 1 [file 41433_2026_4375_MOESM1_ESM.docx]

**Supplementary Table 1: List of participants in the expert panel (listed alphabetically by surname)**

| **Name of participant** | **Speciality and role** | **Institution** |
| --- | --- | --- |
| Sajjad Ahmad | Consultant Ophthalmic Surgeon: Cornea and external eye diseases | Moorfields Eye Hospital NHS Foundation Trust, London; UCL Institute of Ophthalmology, London; Moorfields/UCL NIHR BRC, London |
| Raj Bhayani | Consultant Ophthalmologist | Mid Yorkshire Teaching NHS Trust, West Yorkshire |
| Nicholas Cotton | Consultant Ophthalmologist: Cornea | Royal Victoria Hospital, Belfast |
| Harminder Dua | Professor of Ophthalmology and Visual Sciences | Academic Ophthalmology, Mental Health and Clinical Neurosciences, School of Medicine, University of Nottingham |
| Omar El Haddad | Corneal consultant | Bristol Eye Hospital, University Hospitals Bristol and Weston NHS Foundation Trust;  Faculty of Medicine, Alexandrite University, Egypt |
| Sophie Harper | Principal Optometrist: Cornea | Manchester Royal Eye Hospital, Manchester University NHS Foundation Trust, Manchester |
| Li Jiang | Consultant Ophthalmologist: Cornea, primary care and cataract | Queen Elizabeth Hospital, Birmingham |
| Sai Kolli | Consultant Corneal Surgeon | Queen Elizabeth Hospital, University Hospitals Birmingham NHS Trust & Aston University |
| David Lockington | Consultant Ophthalmologist: Cornea and cataract | Tennent Institute of Ophthalmology, Glasgow |
| Bita Manzouri | Consultant Ophthalmologist: Cataract, cornea and external disease | Queen’s Hospital, BHR University Hospitals NHS Trust, Romford |
| Artemis Matsou | Consultant Ophthalmologist and Cataract Lead | Queen Victoria Hospital, East Grinstead, West Sussex |
| Hasan Naveed | Consultant Ophthalmic Surgeon | Queen Victoria Hospital, East Grinstead, West Sussex |
| Yan Ning Neo | Consultant Ophthalmologist: Cornea | Barts Health NHS Trust, London |
| Michael O’Gallagher | Consultant Ophthalmologist: Cornea | Royal Victoria Hospital, Belfast |
| Liam Price | Consultant Ophthalmologist: Cornea | Portsmouth Hospitals University NHS Trust TBC |
| Haoyu Wang | TSC Fellow: Cornea; Registrar Ophthalmologist | Sheffield Teaching Hospitals NHS Foundation Trust |
