# Supplementary material for: Assessment and management of dry eye disease in the UK: standardising reality-based best practice
Source: Eye (Lond). 2026 Mar 14;40(8):1185–95. doi: 10.1038/s41433-026-04375-7 (PMC13195173; doi:10.1038/s41433-026-04375-7)
Supplement: Supplementary file 2 — Supplementary Table 2 [file 41433_2026_4375_MOESM2_ESM.docx]

**Supplementary Table 2: Additional consensus statements**

| **Statement** | | **Consensus score** | **Percentage who *strongly agree*** | **Percentage who *strongly agree* or *slightly agree*** | **Strength of agreement** |
| --- | --- | --- | --- | --- | --- |
| ***Initial assessment*** | | | | | |
| 7 | Assessment of the above signs with a slit lamp is essential to confirm a diagnosis of DED | **2.93** | **73.3** | **86.7** | **Strong** |
| 11 | **All female patients** should be asked about hormonal changes and medication/supplementation | **2.93** | **46.7** | **100.0** | **Moderate** |
| 12 | Patients with **connective tissue disorders** (such as rheumatoid arthritis) should be considered for hospital referral if they experience DED symptoms and signs | **2** | **40.0** | **80.0** | **Moderate** |
| ***DED subtypes*** | | | | | |
| 13 | Treatment of EDED, ADDED and mixed-subtype DED can vary, but in clinical practice most cases are mixed | **3.87** | **93.3** | **100.0** | **Very strong** |
| 14 | Rapid TBUT, blepharitis and abnormal meibomian glands are indicative of an EDED element | **3.6** | **80.0** | **100.0** | **Very strong** |
| 15 | Low tear volume, reduced tear meniscus and reduced Schirmer's test score are indicative of an ADDED element | **3.6** | **80.0** | **100.0** | **Very strong** |
| ***Initial treatment of DED in the community*** | | | | | |
| 23 | With the right guidance, many patients with mild DED could self-manage their condition | **4.00** | **100.0** | **100.0** | **Very strong** |
| 24 | Patients should be educated by their community optometrist on the correct use of lubricants, warm compresses and massage | **3.73** | **86.7** | **100.0** | **Very strong** |
| 27 | If symptoms are not controlled by twice-daily administration, lubricants should be applied more frequently up to four times daily* | **3.87** | **93.3** | **100.0** | **Very strong** |
| 29 | Greater emphasis should be placed on lid hygiene for patients with EDED, who may also benefit from an oil-based lubricant | **3.60** | **80.0** | **100.0** | **Very strong** |
| 30 | Topical corticosteroids can be initiated in the community by a qualified independent prescribing optometrist | **1.73** | **40.0** | **80.0** | **Weak** |
| 31 | Topical CsA can be initiated in the community by a qualified independent prescribing optometrist | **0.00** | **26.7** | **46.7** | **No consensus** |
| ***Initial treatment of DED in secondary care (general ophthalmology)*** | | | | | |
| 37 | As dosing of topical azithromycin is variable, this should be prescribed by a corneal specialist rather than a general ophthalmologist | **0.93** | **33.3** | **60.0** | **Weak** |
| 39 | Patients with EDED may require an earlier use of doxycycline compared with ADDED | **3.20** | **73.3** | **93.3** | **Very strong** |
| ***Referral*** | | | | | |
| 43 | Patients with filamentary keratitis should be referred to a general ophthalmologist in a **routine** timeframe | **0.93** | **33.3** | **60.0** | **Weak** |
| ***Ongoing management and follow-up*** | | | | | |
| 51 | Once DED is controlled, treatment should adhere to the principle of ‘as little as possible but as much as needed’ | **3.47** | **86.7** | **93.3** | **Very strong** |
| 52 | Lubricants and lid hygiene should be continued for life, even if DED is controlled, but the patient can tailor the frequency to their needs* | **2.53** | **53.3** | **86.7** | **Strong** |
| 53 | Doxycycline should be held in reserve for rescue in case of flare-up | **2.00** | **26.7** | **86.7** | **Weak** |
| 54 | If DED is controlled by CsA, ongoing treatment should align with current guidelines | **3.73** | **86.7** | **100.0** | **Very strong** |
| ***Discharge*** | | | | | |
| 57 | Patients should be made aware of the nature of their disease, how to manage it, when to seek help and a point of contact in case of flare-ups | **3.73** | **86.7** | **100.0** | **Very strong** |
| 58 | Patients with DED could be discharged from secondary care to the care of their optometrist or GP for maintenance | **3.73** | **86.7** | **100.0** | **Very strong** |
| 61 | Patients previously treated for moderate DED who experience a recurrence of symptoms, if not in a PIFU scheme, should initially present to their community optometrist | **2.67** | **60.0** | **86.7** | **Strong** |
| 62 | Patients previously treated for moderate DED who experience a recurrence of symptoms, if not in a PIFU scheme, should initially present to their community optometrist, or to the hospital eye service if their symptoms are severe | **3.73** | **86.7** | **100.0** | **Very strong** |

*Statement applies to both eyes.
