# Supplementary material for: Assessment and management of dry eye disease in the UK: standardising reality-based best practice
Source: Eye (Lond). 2026 Mar 14;40(8):1185–95. doi: 10.1038/s41433-026-04375-7 (PMC13195173; doi:10.1038/s41433-026-04375-7)
Supplement: Supplementary file 3 — Supplementary Table 3 [file 41433_2026_4375_MOESM3_ESM.docx]

**Supplementary Table 3: Frequency of follow-up**

| **Frequency of follow-up** | **Patients seen at this frequency** | **Responsible Eye Health Professional** |
| --- | --- | --- |
| **Weekly** | Red-flag issues and urgent referrals | Corneal specialist |
| **Monthly** | Severe DED at the beginning of treatment; non-responders to treatment | Corneal specialist |
| **Three-monthly** | Patients receiving short-course topical corticosteroids and recently initiated on treatment: monitoring for treatment side-effects | General ophthalmologist |
| **Six-monthly** | DED responding to treatment: symptoms improving, signs resolving, no/manageable treatment side-effects | Discharge to care of local optometric practitioner (or IP optometrist if the patient is on ongoing prescribed treatment, such as ciclosporin) |
| **Discharge** | DED under control: symptoms tolerable, signs resolving or resolved | General ophthalmologist/ optometrist |
