# Supplementary material for: Assessment and management of dry eye disease in the UK: standardising reality-based best practice
Source: Eye (Lond). 2026 Mar 14;40(8):1185–95. doi: 10.1038/s41433-026-04375-7 (PMC13195173; doi:10.1038/s41433-026-04375-7)
Supplement: Supplementary file 4 — Supplementary Table 4 [file 41433_2026_4375_MOESM4_ESM.docx]

**Supplementary Table 4: Overview of strengths and weaknesses of different assessments for DED**

| **Assessment** | **Strengths** | **Weaknesses** |
| --- | --- | --- |
| **Schirmer’s Test** | - Important for assessment of Sjogren’s Syndrome - Assesses volume of the tear film *(Wolffsohn JS, et al. Am J Ophthalmol 2025;279:387–450; Wolffsohn JS, et al. Ocul Surf 2017;15(3):539–74)* | - Not all practitioners have access to a Schirmer’s strip - Poor reproducibility *(Wolffsohn JS, et al. Am J Ophthalmol 2025;279:387–450; Senchyna M, Wax MB. J Ocul Biol Dis Infor 2008;1(1):1–6)* - Long time required for testing *(Senchyna M, Wax MB. J Ocul Biol Dis Infor 2008;1(1):1–6)* - Invasive *(Wolffsohn JS, et al. Am J Ophthalmol 2025;279:387–450)* - Variable testing methods *(Senchyna M, Wax MB. J Ocul Biol Dis Infor 2008;1(1):1–6)* |
| **Tear Meniscus Height** | - Most direct approach to study the volume of the tear film *(Wolffsohn JS, et al. Ocul Surf 2017;15(3):539–74)* - Can be measured with the height of the slit beam on the slit lamp *(Wolffsohn JS, et al. Am J Ophthalmol 2025;279:387–450)* | - Poor reproducibility *(Wolffsohn JS, et al. Am J Ophthalmol 2025;279:387–450)* - Subjective without specialised equipment *(Wolffsohn JS, et al. Ocul Surf 2017;15(3):539–74)* |
| **Meibography** | - Allows visualisation of changes in meibomian gland morphology *(Wolffsohn JS, et al. Ocul Surf 2017;15(3):539–74)* | - Requires specialised equipment *(Wolffsohn JS, et al. Ocul Surf 2017;15(3):539–74)* - Diagnostic value not yet established *(Wolffsohn JS, et al. Ocul Surf 2017;15(3):539–74)* |
| **Tear Osmolality** | - Least variable of the common DED signs *(Wolffsohn JS, et al. Ocul Surf 2017;15(3):539–74)* - Generally increases with disease severity *(Wolffsohn JS, et al. Ocul Surf 2017;15(3):539–74)* | - Requires specialised equipment *(Wolffsohn JS, et al. Am J Ophthalmol 2025;279:387–450)* - Daily variation in osmolarity is the best marker but impractical in a clinical setting *(Wolffsohn JS, et al. Am J Ophthalmol 2025;279:387–450)* |
| **Corneal sensation** | - Lack of sensation is a major red flag for neurotrophic keratitis *(Stolz M. Clin Ophthalmol 2025;19:1323–30)* - Impaired corneal sensation can lead to worsened symptoms and signs of DED *(Stolz M. Clin Ophthalmol 2025;19:1323–30)* | - Aesthesiometer required for objective measurement *(Wolffsohn JS, et al. Am J Ophthalmol 2025;279:387–450)* |
