# Supplementary material for: Assessment and management of dry eye disease in the UK: standardising reality-based best practice
Source: Eye (Lond). 2026 Mar 14;40(8):1185–95. doi: 10.1038/s41433-026-04375-7 (PMC13195173; doi:10.1038/s41433-026-04375-7)
Supplement: Supplementary file 5 — Supplementary Box 1 [file 41433_2026_4375_MOESM5_ESM.docx]

**Supplementary Box 1: Common medications linked to DED**

- Psychoactive medications, such as anxiolytics and antidepressants
- Hormonal therapy, such as HRT and anti-androgen therapy
- Certain dermatological medications, such as isotretinoin
- Antihistamines (and other anticholinergic medications such as antiparkinsonian and anti-arrhythmic agents)

*Sheppard J, et al. Ann Med 2023;55:241–252; Stapleton F, et al. Am J Ophthalmol 2025;279:451–553.*
