# Supplementary material for: Assessment and management of dry eye disease in the UK: standardising reality-based best practice
Source: Eye (Lond). 2026 Mar 14;40(8):1185–95. doi: 10.1038/s41433-026-04375-7 (PMC13195173; doi:10.1038/s41433-026-04375-7)
Supplement: Supplementary file 6 — Supplementary Box 2 [file 41433_2026_4375_MOESM6_ESM.docx]

**Supplementary Box 2: Overview of less commonly available procedures in the UK**

**Lipiflow**

Medical device that provides heat and pressure to meibomian glands in order to liquefy hardened meibum; intended to be more consistent and reliable compared with a warm compress. Application of topical anaesthesia is required before using. Data is limited.

*(Jones L, et al. Am J Ophthalmol 2025;279:289–386; Pucker AD, et al. Cochrane Database Syst Rev 2024:CD015448)*

**Intense pulsed light (IPL)**

Broad-wavelength light to stimulate blood coagulation in superficial blood vessels: precise mechanism of action is unclear. Initially used to treat skin conditions such as rosacea. Generally targets the skin below the lower eyelid and both temporal regions, but not the upper eyelid. Data for DED is limited but a recent RCT found improvements in TBUT when IPL was combined with meibomian gland expression.

(*Cote S, et al. Cochrane Database Syst Rev 2024:* *CD013559; Jones L, et al. Am J Ophthalmol 2025;279:289–386; Toyos R, et al. PLoS ONE 2022;17:e0270268)*

**Meibomian probing**

Insertion of a sterile probe to remove blockages in meibomian glands. Data is limited and efficacy is unclear; only short-term safety data are available.

*(Jones L, et al. Am J Ophthalmol 2025;279:289–386; Magno M, et al. Surv Ophthalmol 2021;66:612–622)*
