# Supplementary material for: Assessment and management of dry eye disease in the UK: standardising reality-based best practice
Source: Eye (Lond). 2026 Mar 14;40(8):1185–95. doi: 10.1038/s41433-026-04375-7 (PMC13195173; doi:10.1038/s41433-026-04375-7)
Supplement: Supplementary file 7 — Supplementary Figure 1 [file 41433_2026_4375_MOESM7_ESM.pptx]

## Slide 1
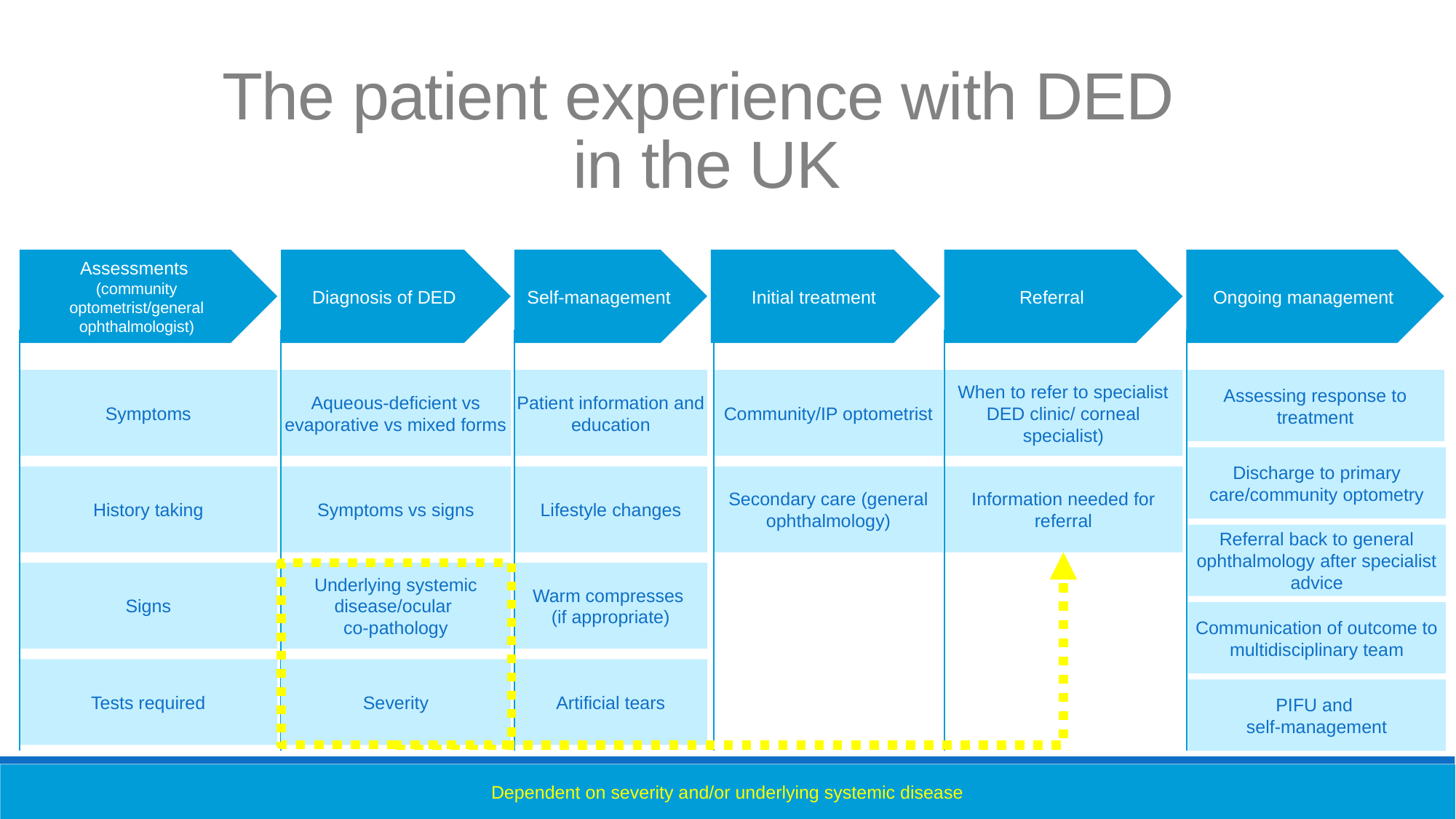

The patient experience with DED
in the UK
Assessments
(community optometrist/general ophthalmologist)
Diagnosis of DED
Self-management
Initial treatment
Referral
Ongoing management
Symptoms
Aqueous-deficient vs evaporative vs mixed forms
Patient information and education
Community/IP optometrist
When to refer to specialist DED clinic/ corneal specialist)
Assessing response to treatment
Discharge to primary care/community optometry
History taking
Symptoms vs signs
Lifestyle changes
Secondary care (general ophthalmology)
Information needed for referral
Referral back to general ophthalmology after specialist advice
Signs
Underlying systemic disease/ocular
co-pathology
Warm compresses
(if appropriate)
Communication of outcome to multidisciplinary team
Tests required
Severity
Artificial tears
PIFU and
self-management
Dependent on severity and/or underlying systemic disease
IP, independent prescriber; PIFU, patient-initiated follow-up.
