# Supplementary material for: Assessment and management of dry eye disease in the UK: standardising reality-based best practice
Source: Eye (Lond). 2026 Mar 14;40(8):1185–95. doi: 10.1038/s41433-026-04375-7 (PMC13195173; doi:10.1038/s41433-026-04375-7)
Supplement: Supplementary file 8 — Supplementary Figure 2 [file 41433_2026_4375_MOESM8_ESM.pptx]

## Slide 1
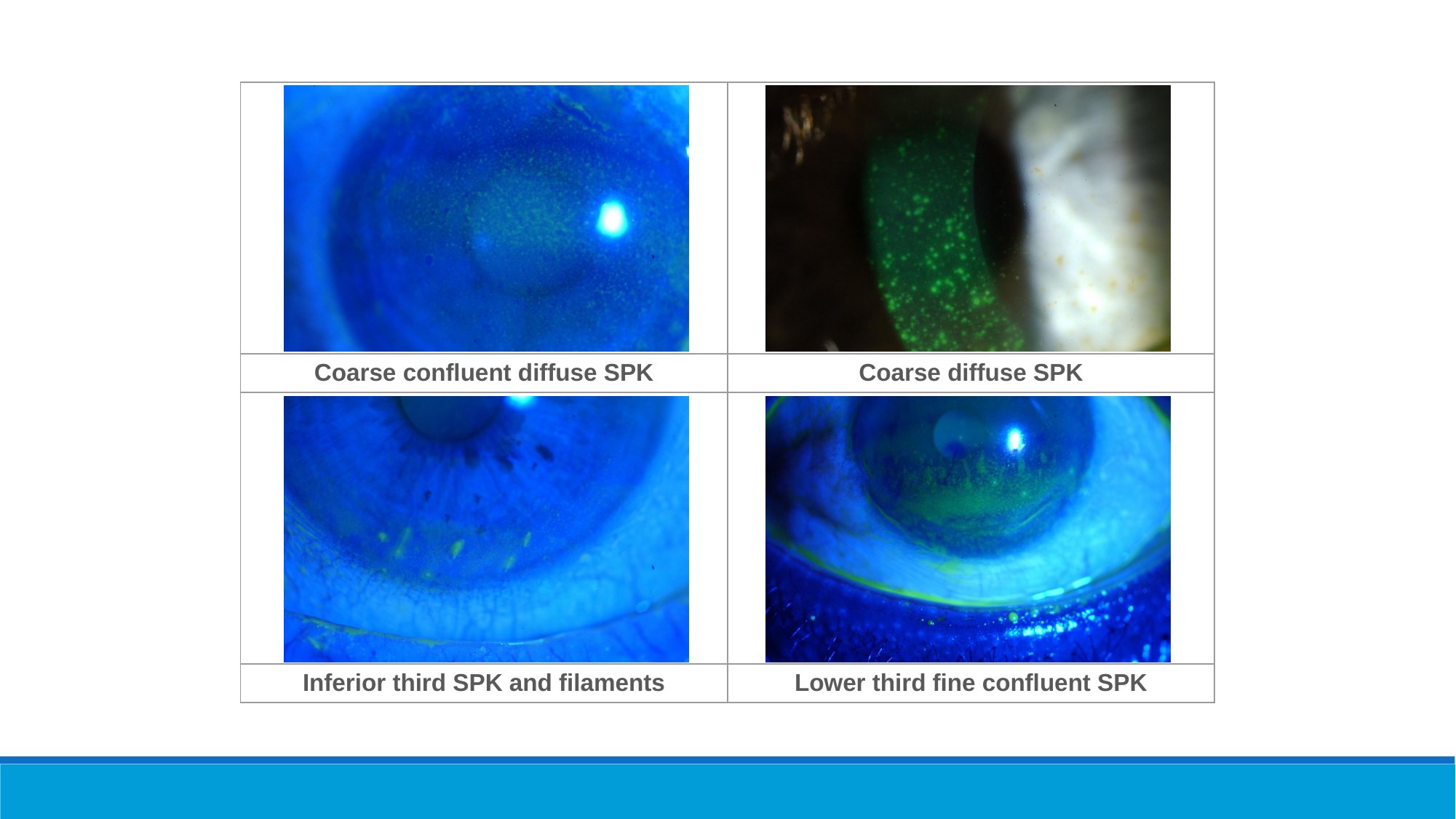

| | |
| --- | --- |
| Coarse confluent diffuse SPK | Coarse diffuse SPK |
| | |
| Inferior third SPK and filaments | Lower third fine confluent SPK |
